# A Different Brain: Anomalies of Functional and Structural Connections in Williams Syndrome

**DOI:** 10.3389/fneur.2018.00721

**Published:** 2018-09-13

**Authors:** Chiara Gagliardi, Filippo Arrigoni, Andrea Nordio, Alberto De Luca, Denis Peruzzo, Alice Decio, Alexander Leemans, Renato Borgatti

**Affiliations:** ^1^Neuropsychiatry and Neurorehabilitation Unit, Scientific Institute, IRCCS E. Medea, Bosisio Parini, Italy; ^2^Neuroimaging Lab, Scientific Institute, IRCCS E. Medea, Bosisio Parini, Italy; ^3^Department of Information Engineering, University of Padova, Padova, Italy; ^4^Image Sciences Institute, University Medical Center Utrecht, Utrecht, Netherlands

**Keywords:** williams syndrome, DTI, fMRI, connectivity, rehabilitation

## Abstract

We describe the results of a functional and structural brain connectivity analysis comparing a homogeneous group of 10 young adults with Williams Syndrome (WS; 3 females, age 20. 7 ± 3.7 years, age range 17.4–28.7 years) to a group of 18 controls of similar age (3 females, age 23.9 ± 4.4 years, age range 16.8–30.2), with the aim to increase knowledge of the structure – function relationship in WS. Subjects underwent a 3T brain MRI exam including anatomical, functional (resting state) and structural (diffusion MRI) sequences. We found convergent anomalies in structural and functional connectivity in the WS group. Altered Fractional Anisotropy (FA) values in parieto-occipital regions were associated with increased connectivity in the antero-posterior pathways linking parieto-occipital with frontal regions. The analysis of resting state data showed altered functional connectivity in the WS group in main brain networks (default mode, executive control and dorsal attention, sensori-motor, fronto—parietal, ventral stream). The combined analysis of functional and structural connectivity displayed a different pattern in the two groups: in controls the highest agreement was found in frontal and visual areas, whereas in WS patients in posterior regions (parieto-occipital and temporal areas). These preliminary findings may reflect an altered “wiring” of the brain in WS, which can be driven by hyper-connectivity of the posterior regions as opposed to disrupted connectivity in the anterior areas, supporting the hypothesis that a different brain (organization) could be associated with a different (organization of) behavior in Williams Syndrome.

## Introduction

William Syndrome (WS) is a neurodevelopmental disorder caused by a hemizygous deletion in 7q11.23 (1). In most cases the deletion involves approximately 1.55 megabases (Mb) encoding 26–28 genes in the WS critical region (WSCR); less often (approximately in 5% of cases) a slightly larger deletion of 1.84 Mb occurs (2). In addition, other rare atypical deletions have been reported, highlighting the role of specific genes in the clinical phenotype, mainly as to visual spatial and social competences (3–5). WS has a prevalence of 1:7.500 to 20.000 live births (6, 7) and is characterized by mild-to-severe intellectual disability and an uneven social and cognitive profile (8).

The neurocognitive profile of WS is characterized by a peculiar impairment in visuospatial construction with a relative preservation of concrete language skills, and an overall mild- to- severe intellectual disability (8, 9). Individuals with WS usually display high sociability, excessive empathy, impulsivity, inattention, sadness, and depression as well as generalized anxiety disorder and hyperactivity disorder (ADHD) (10).

In recent years WS has been considered an “experiment of nature” (11, 12) that could help to shed a light on the genotype/phenotype relations as well as on the links between behavior and brain structures (13). The genetic features and the peculiar imbalance in cognitive and social competences, with uneven development in both domains, have been associated with a series of structural and functional brain abnormalities mainly in visual spatial, executive, and social processing networks, reported both in children and adults with WS by using sophisticated structural and functional neuroimaging approaches (14). Structural studies documented that the volumes of frontal and temporal regions, as well as of the cerebellar cortex are relatively preserved, whereas volumes of parietal and occipital cortices and of the basal ganglia are markedly reduced (15, 16). The overall reduction in brain volume in WS is thought to be due to an early reduction in surface area, whereas anomalous development (delay or arrest) could explain an increased cortical thickness with respect to controls (17) and an overall unusual shape in adult age linked to brain structural alterations of the posterior cerebrum (18). Recently Fan et al. (19) have described abnormalities in the cortical-subcortical circuitry—mainly orbitofrontal cortex, superior parietal cortex, Sylvian fissure and basal ganglia—in young and adult WS, related to their cognitive and behavioral profile.

With diffusion tensor imaging (DTI), previous research has found modifications in white matter tracts orientation and reduced lateralization of fiber coherence, putatively due to a late alteration of the migration process (20). As to functional MRI (fMRI) studies, most research has focused on task related modifications and less on resting state functional connectivity. Among the latter, (21) and (22) focused on the default mode network (DMN), a group of well-established areas that, among many functions, are also involved in self-referential function as well as emotional and cognitive processing and in social cognition. Vega et al. (21) described an altered connectivity in the DMN in WS participants compared to healthy controls (HC) while (22) found a decreased functional connectivity (FC) in a posterior hub of the DMN including the precuneus, calcarine and the posterior cingulate of the left hemisphere.

Though separately well described, the regional features of structural and functional brain networks are rarely assessed at the same time in WS. The simultaneous study of the structural and functional connectivity underlying the brain networks could help in understanding the pathophysiological mechanisms and the developmental trajectories of the behavioral manifestations in WS syndrome, as implied in the concept of the “connectome,” the structured description of the elements forming the human brain and the connections between them (23, 24).

The aim of our preliminary study is twofold: 1) we want to assess if simultaneous evaluation of functional and structural connectivity is feasible in WS patients and 2) given the hypothesis of an altered “wiring” of the brain in WS, we want to confirm or highlight the presence of (new) altered brain regions or circuits associated with the neurocognitive profile of the syndrome, with a comparative and integrate analysis of two different imaging modalities (fMRI and DTI).

## Materials and methods

### Participants

The study reports data collected from 10 participants with Williams syndrome (3 females, age 20.7 ± 3.7 years, age range 17.4–28.7 years) and from 18 HC (3 females, age 23.9 ± 4.4 years, age range 16.8–30.2). All patients carried a typical 7q11.23 deletion, confirmed using fluorescent *in situ* hybridization testing and underwent cognitive evaluation conducted by trained psychologists using the age-appropriate versions of the Wechsler Scales. [WAIS R, (25)]. The presence of minor psychiatric symptoms, mainly focusing on anxiety (9, 26), was assessed as in Vicari et al. (10), both by interviews of patients and patients' caregivers performed by an expert clinician and by the semi-structured psychiatric diagnostic interview “Kiddie-Sads-Present and Lifetime Version” (K-SADS-PL) (27, 28). WS participants had no history of past neurological disorders or sensorial loss.

HCs had no history of psychiatric or neurologic illness, learning disabilities, or hearing or visual loss. They showed average school performances in language and reading, and had an intelligence quotient (IQ) of at least 85 on the Cattell's Culture Fair Intelligence Test (29). Emotional and behavioral problems in HC were assessed by CBCL the Child Behavioral Checklist and 18 Youth Self Report or Adult Behavior Checklist (30). None of HC exceeded the clinical cutoff in the Total Problems Scale or in subscales (internalizing and externalizing problems, ADHD or pervasive developmental disorder).

Participants with WS were recruited via proposal both to the Association of Family of persons with Williams Syndrome and to the patients followed up at E.Medea Research Institute. Typically developing controls were recruited through parents' networks and panel advertisement at the local University. Exclusion criteria for all groups included premature birth (gestational age under 34 weeks), known diagnosis of a major psychiatric disorder, including psychotic or mood disorders, or current neurological disorder including seizures, and any contraindications from the MRI scan. Demographic details of the entire group are shown in Table 1. Both patients and controls were part of an ongoing research project on MRI in neurodevelopmental disorders, in accordance with the recommendations of E. Medea Ethics Committee. All subjects or they legal guardian gave written informed consent in accordance with the Declaration of Helsinki. The protocol was approved by the E. Medea Ethics Committee on 11-3-2014.

**Table 1:** Demographic characteristics of all participants with WS.

**Patient**	**Verbal IQ**	**Perfomance IQ**	**Full IQ**	**Anxiety**
1	69	73	68	2
2	56	53	57	2
3	58	50	51	1
4	65	60	61	2
5°	71	59	57	3
6	68	51	56	3
7*	63	52	52	3
8	50	47	44	3
9	51	45	44	3
10	68	72	67	3
Mean (sd)	61,9 (7,7)	56,2 (9,8)	55,7 (8,3)	

In Anxiety column, class 3 represents “clinically significant”, 2 expresses a trend, and 1 the absence of clinical symptoms as by K SADS. Mean values are in bold and standard deviation in brackets. Participant excluded from the analysis of ^*^functional and °structural connectivity are evidenced.

### MRI protocol

Patients underwent a 3T brain MRI exam with a Philips Achieva dStream scanner at E. Medea Research Institute. The acquisition protocol included anatomical, functional (resting state) and structural diffusion MRI (dMRI) sequences for DTI quantification and connectomics. A high resolution T1-weighted MPRAGE sequence (voxel resolution 1 × 1 × 1mm^3^, matrix size 256 × 256 × 160, TE/TR 3.86/8.40 ms, SENSE 2) was acquired as anatomical reference. A T2^*^-weighted single-shot echo-planar imaging (EPI) sequence was used (voxel resolution 2.5 × 2.5 × 4mm^3^, matrix size 96 × 96 × 30, TR/TE = 2000/30 ms, 250 dynamic scans, flip angle = 90°) for fMRI with subjects keeping their eyes closed. Diffusion MRI data were acquired using a two-shell T2-weighted single-shot EPI sequence (voxel resolution 2.2 × 2.2 × 2.2 mm^3^, matrix size 112 × 112 × 80, TE/TR 100/8800 ms, SENSE 2), featuring 78 volumes with the PA phase encoding (9 b = 0s/mm^2^, 16 directions at b = 300s/mm^2^, 53 directions at b = 1100s/mm^2^) and 13 volumes (7 b = 0s/mm^2^, 6 directions at b = 1100s/mm^2^) acquired with the AP phase encoding for a better correction of EPI distortions. A T2-weighted Turbo Spin Echo sequence (voxel resolution 1.5 × 1.5 × 1.5mm^3^, matrix size 160 × 146 × 110, TE/TR 100/4700 ms, SENSE 2, SPIR fat suppression) was also acquired for the post-processing of functional and dMRI data. The duration of the scanning session was approximately 37 min.

HC were scanned with the same protocol including T1-weighted, T2-weighted and fMRI sequences. However, the same dMRI acquisition was only available in 10 HC.

### dMRI processing and analysis

One patient (patient 5) showed corrupted dMRI data (movement artifacts) and was discarded from the following analysis. Diffusion data of 9 subjects (mean age 20.4 years, IQ 55.6) were pre-processed with the DIFFPREP module of TORTOISE (31) to remove motion-related and eddy current induced artifacts. In addition, correction of geometrical EPI distortions was performed with DR-BUDDI using the dual phase encoding acquisition. Corrected data were fitted with the DTI model using the RESTORE non-linear least squares estimator as part of TORTOISE, then the diffusion tensor was exported to the DTI-TK format. The T1-weighted data and the partial volume maps were rigidly registered with ANTS to the corrected data using the T2-weighted image as an intermediate target (32).

A study-specific template was built with the tensor-based registration included in DTI-TK, then the individual tensors of the subjects were moved to the atlas space. Fractional anisotropy (FA) maps were computed from the transformed tensors to perform voxel-wise statistics with FSL RANDOMIZE. The statistical analysis featured the TFCE correction with a critical value of 0.05 corrected for multiple comparisons.

Following the voxel-wise statistics, a structural connectivity analysis was performed for each dataset. Only the dMRI data acquired at b = 0 s/mm^2^ and b = 1100s/mm^2^ were used at this stage. The first part of the connectivity analysis consisted in the creation of the individual tractogram, a step that we performed with MRTrix 3 (v 313) using the Constrained Spherical Deconvolution (CSD) approach. Even though CSD is generally performed with diffusion data acquired at b-value higher than 1100s/mm^2^, this approach is still viable and reduces the burden of crossing fibers compared to the DTI based fiber-tracking. The response function was computed using an FA threshold equal to 0.7 and a spherical harmonics order equal to 4, then CSD was performed on the data of each subject to reconstruct the corresponding fiber orientation distribution (FOD).

Fiber-tracking was performed using the iFOD 2 method and the default/optimized tracking parameters. Twenty million streamlines were generated for each subject using the gray matter/white matter (GM/WM) interface (derived with MRtrix from the GM/WM T1-weighted segmentation) as seeding and the simultaneous Anatomically-Constrained Tractography (ACT) correction. The GM/WM seeding has been shown to reduce the influence of fiber bundles size on the final track density (33), while ACT ensures only anatomically feasible tracks are retained, i.e., tracks that connect two GM areas through WM. Therefore, spurious tracks terminating into cerebrospinal fluid, into WM or never leaving GM were not included in the tractography result. After the fiber-tracking step, the tractogram was filtered with the SIFT technique, pruning 10M of the 20M streamlines, obtaining the final 10M streamlines tractogram. The SIFT filtering ensures the match between the voxel-wise FOD and the streamlines count through each voxel.

The Automated Anatomical Labeling—AAL atlas was non-linearly registered to the diffusion space of each subject with ANTS using the T1-weighted image as intermediate target, then connectivity matrices of each subject were built assigning the tracks to each node with a local search algorithm (maximum search radius 2 mm). For the statistical analysis of the resulting connectome the NBS package was employed to perform two-samples one-sided *t*-tests. Tests were corrected for multiple comparisons with FDR using a critical threshold equal to 0.05 and 50,000 permutations.

Correlation between the connection probability and the behavioral measures was assessed with Pearson correlations. The tests were performed only between the pair of nodes found to be different in the abovementioned comparative analysis between HCs and WS patients, and were considered significant at 0.05 Bonferroni corrected.

### fMRI processing and analysis

One patient (patient 7) showed corrupted FMRI data (movement artifacts) and was discarded from the following analysis. T1-weighted and T2-weighted data from 9 patients (mean age 20.9 years, IQ 56.1) were then processed with the N4 tool of ANTS to mitigate the intensity inhomogeneity bias, then skull removal was performed with the FSL tool BET. Functional volumes were slice time corrected with SPM12 and realigned with MCFLIRT of FSL. The average volumes of the resting-fMRI realigned sequences were registered to a common anatomical space by a three-step procedure. (1) non-linear registration of the average fMRI to the corresponding skull-stripped T2-weighted sequence; (2) rigid registration between T2-weighted and T1-weighted sequences; (3) affine and non-linear registration between T1-weighted and MNI reference.

To achieve a better match between anatomical structures between subjects in the standard space, a study template was created averaging the subjects' mean functional EPI in MNI space: this template was used as a reference for the second registration between EPI in the native space and the template in the MNI space. The mean signal in cerebrospinal fluid, in the white matter, the signal drift and motion parameters obtained during the realignment step were regressed out in the native space, then a band-pass temporal filter (0.008–0.1 Hz) was applied to remove physiological and non-BOLD related effects. Functional sequences were then normalized (with voxels resampled at 3 mm^3^) and smoothed with a Gaussian spatial filter (FWHM = 5 mm).

Group analysis was conducted by applying independent component analysis (ICA) as implemented in FSL MELODIC software, by multi-session temporal concatenation. An automatic procedure estimation was used to select 24 ICA group-components. The selection of “good” and “bad” components (i.e., components related to neural activity and components related to noise/artifacts), was done by visual inspection of the spatial patterns of ICA maps, and the power spectrum of the time courses. This procedure led us to label 16 components as related to resting state networks (RSNs). Using the dual regression method (34), we used this set of group-average spatial maps to generate a corresponding set of subject-specific spatial maps and time-series for each subject. We then obtained one spatial ICA map and one time signal for each subject and each group-ICA component. These spatial maps and time-series were then fed to further analysis.

We tested for group differences in intra-network functional connectivity (FC), i.e., how brain regions are more or less involved into a specific network, using FSL's randomize permutation test tool, applied to the subject-specific ICA-specific spatial maps. For the intra-network FC analysis we adopted a threshold of *p* < 0.05 (corrected for multiple comparisons).

Correlation between functional connectivity in those network that appeared to be altered in WS patients and behavioral measures was tested using RANDOMIZE implemented in FSL (*p* < 0.05 corrected for multiple comparison).

### Functional and structural connectivity agreement

The method proposed by Horn et al. (35) was used to evaluate the relationship between functional and structural connectivity. Due to the small sample size, we calculated functional and structural connectivity matrix on a ROI-based level, using the AAL cortical regions, excluding the cerebellum. For the functional connectivity, we computed the mean temporal signal of the preprocessed functional data, for each subject and ROIs. Then, the functional connectivity matrix was computed using the Pearson's correlation between the average signals of the ROIs, resulting in N × N matrix for each subject. Structural connectivity was calculated as described in the previous section.

Pearson's correlation was used, to compute the agreement matrix between functional and structural connectivity data (35). This pairwise analysis involved 8 WS subjects and 7 HC subjects.

## Results

### Diffusion MRI

**Figure 1:**
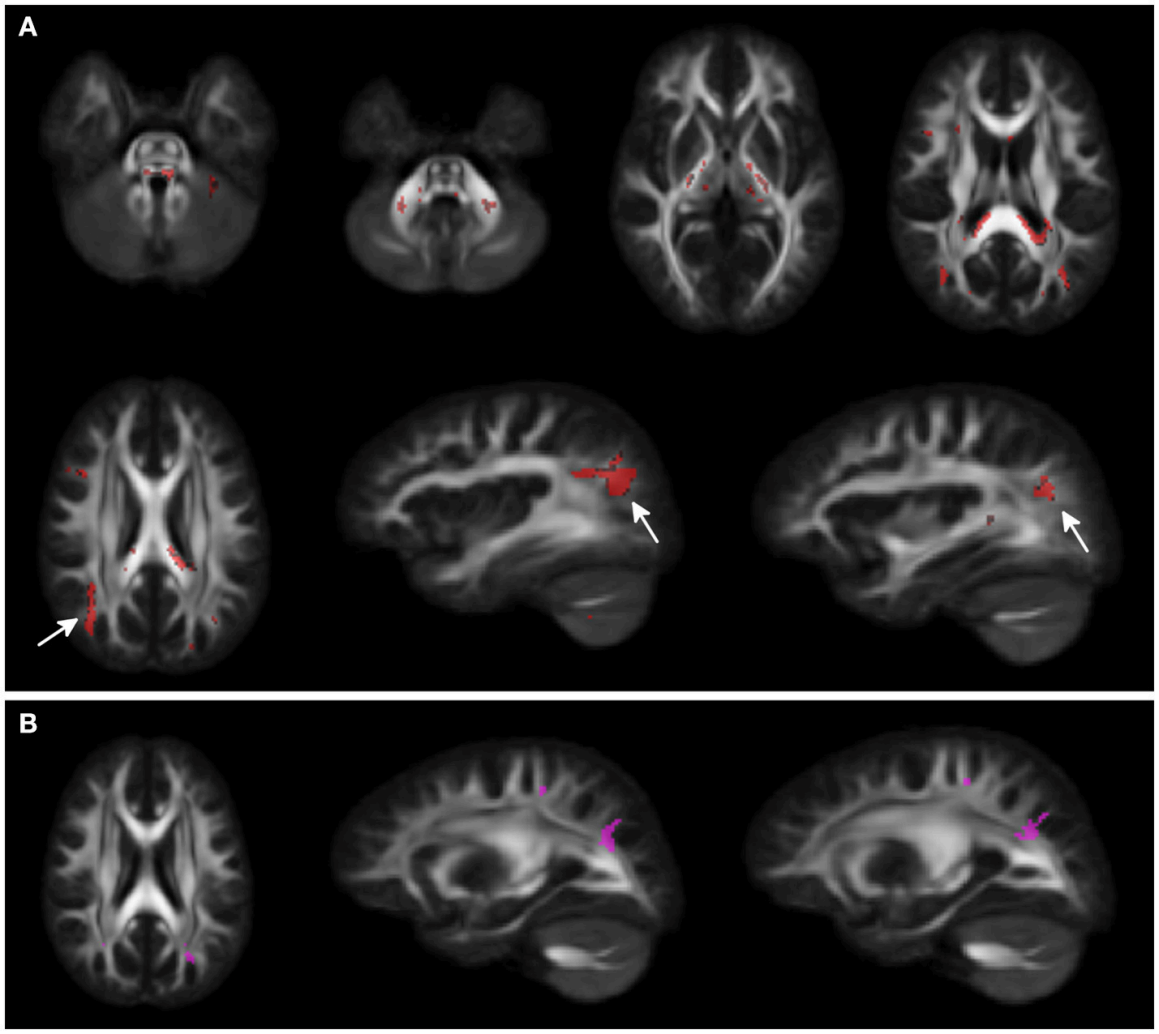
Voxel-wise Fractional anisotropy (FA) differences between Williams Syndrome (WS) and healthy controls (HC). Panel **(A)** (First 2 rows) shows in red the areas where FA values are lower in WS patients compared to the HCs. Arrows point at bilateral parieto-occipital clusters. Panel **(B)** shows the small cluster of higher FA in the left hemisphere (arrowheads) for the WS group compared to the HCs. Significance level is set at *p* < 0.05 corrected for multiple comparisons.

Voxel-wise analysis showed clusters of lower FA in middle and superior cerebellar peduncles, posterior limbs of internal capsules, and in the splenium of corpus callosum for WS patients compared to HCs. A larger cluster of lower FA was observed in the subcortical white matter of the parieto-occipital region bilaterally. A small cluster of higher FA was present in the adjacent parieto-occipital white matter of the left hemisphere, in WS patients compared to HCs (Figure 1).

**Figure 2:**
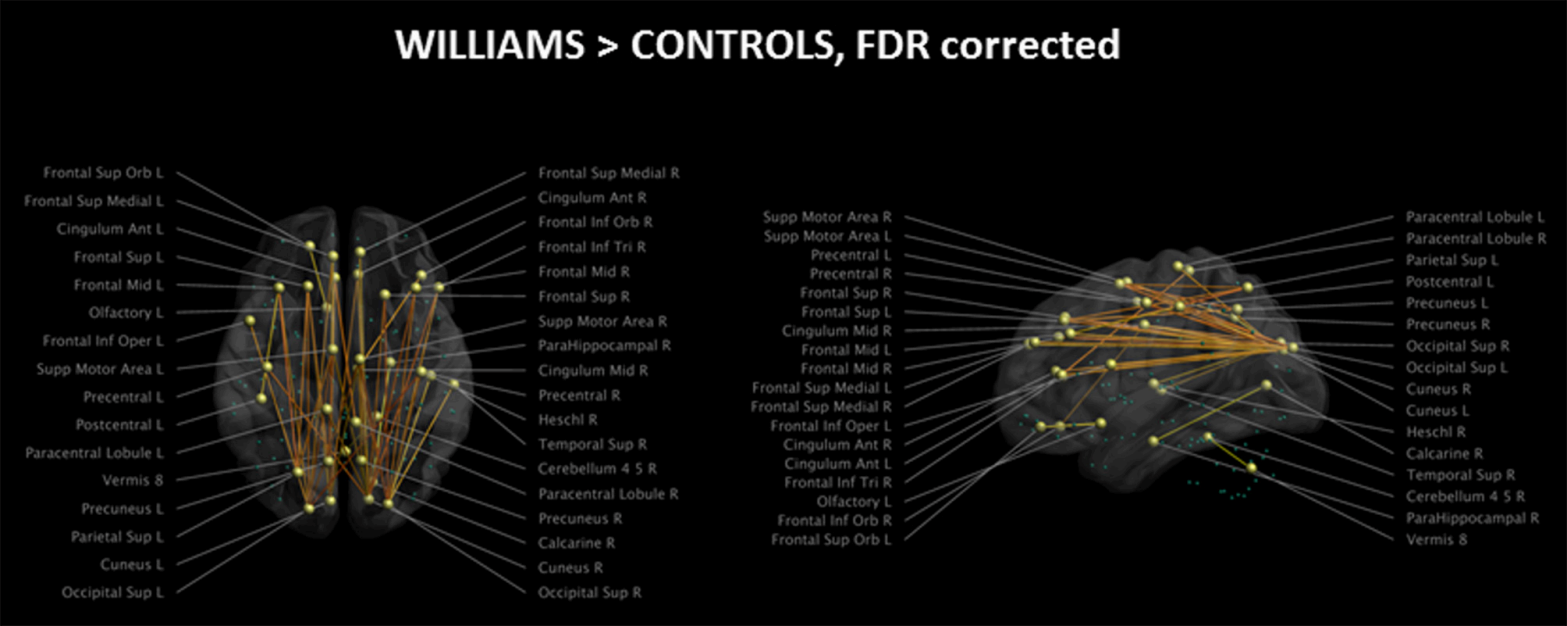
Impaired structural connectivity in WS patients compared to HCs (left: axial view; right: sagittal view). Most of the impaired connections originate from parieto-occipital regions and are directed to frontal areas. On the axial view, it is evident that very few transcallosal connections are impaired. Green spheres represent nodes with no anomalies in structural connectivity. Significance level is set at *p* < 0.05 corrected for multiple comparisons.

The analysis of the structural connectome is shown in Figure 2, while the detailed list of statistically different connections is reported in Supplementary Table 1. WS patients showed a higher degree of connections between few parieto-occipital areas of both hemispheres (Cuneus, Precuneus, and Superior Occipital cortex) and frontal areas, cingulum, and parahippocampus (Figure 2). Most of these differences were located in the same hemisphere, whereas only a few of them were transcallosal. Other differences in connectivity could be detected in the frontal lobe, fronto-parietal areas, orbital cortex, temporo-occipital areas, and cerebellum.

Of the 116 cortical nodes we tested, only 34 showed differences in connectivity, all showing an antero-posterior axis. We did not observe differences in connections between homotopic regions of the two hemispheres or along the supero-inferior axis of the brain.

### fMRI

**Figure 3:**
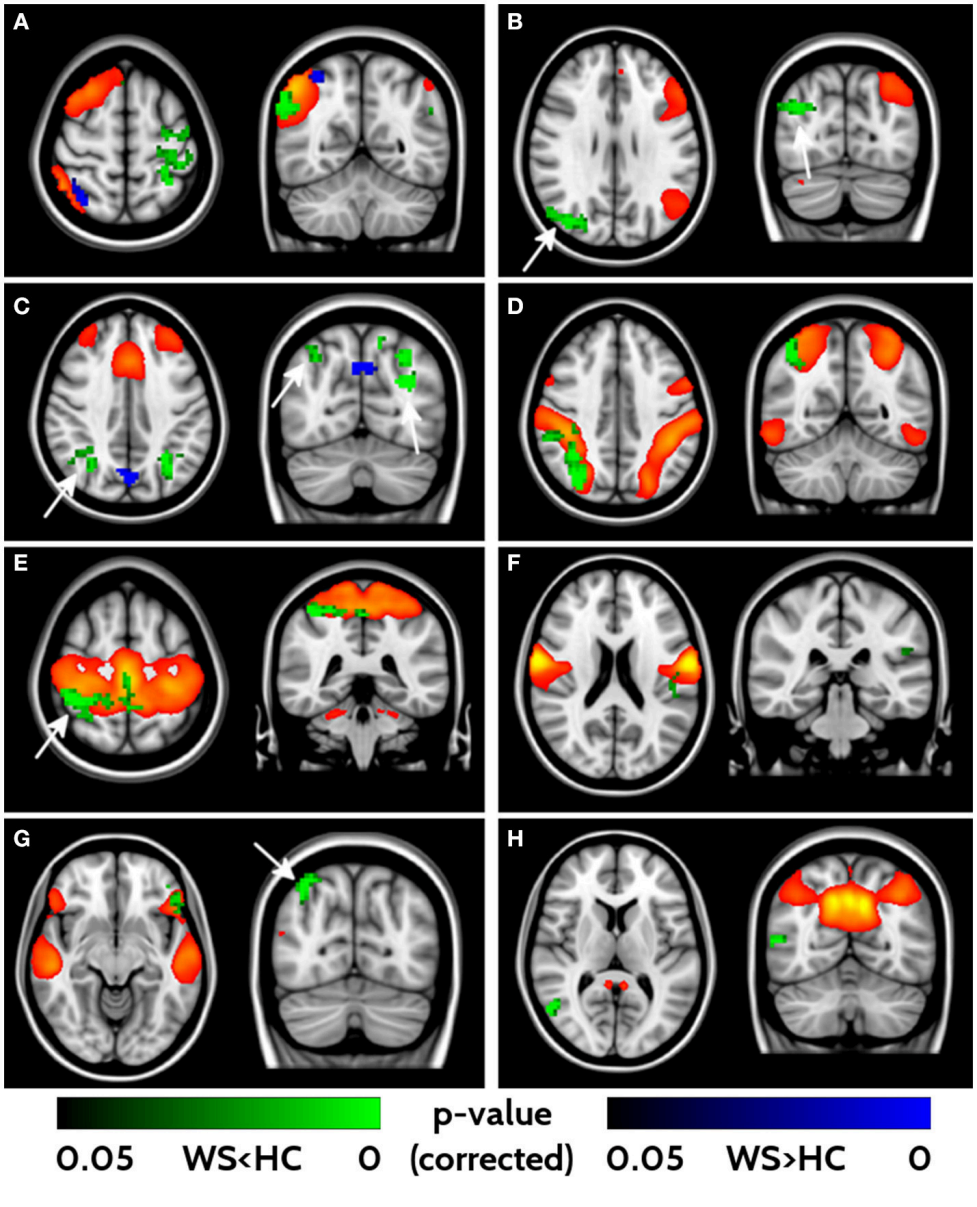
Impaired functional connectivity in WS patients. Right- and left-frontoparietal networks **(A, B)**, executive control network **(C)**, dorsal attention network **(D)**, sensorimotor networks **(E, F)**, ventral stream **(G)** and default mode networks **(H)** showed significant differences in functional connectivity between WS and HCs. Yellow/red voxels represent the areas involved in each network. Areas of lower functional connectivity (FC) in WS patients compared to HCs are shown in green, whereas areas of higher FC in WS patients compared to HCs are shown in blue. Arrows point at bilateral parieto-occipital clusters characterized by impaired FC. Significance level is set at *p* < 0.05 corrected for multiple comparisons.

With the fMRI analysis, we tested for group differences in FC within the 16 networks identified with ICA. Eight out of sixteen components revealed different FC between WS patients and HCs (Figure 3). All the significant networks (right and left fronto-parietal, executive control, dorsal attention, anterior, and posterior sensori-motor, ventral stream and default mode networks) showed areas of lower FC in WS patients compared to HC, with only two (right fronto-parietal and executive control networks) showing also small clusters of higher connectivity in the WS patients group. Areas of lower connectivity for WS patients were mainly located in bilateral parieto-occipital lobes (Cuneus, Precuneus and Superior Occipital cortex) and posterior frontal cortex. Smaller areas of lower connectivity were also located in ventrolateral prefrontal cortex and lateral parietal cortex.

### Functional and structural connectivity agreement

**Figure 4:**
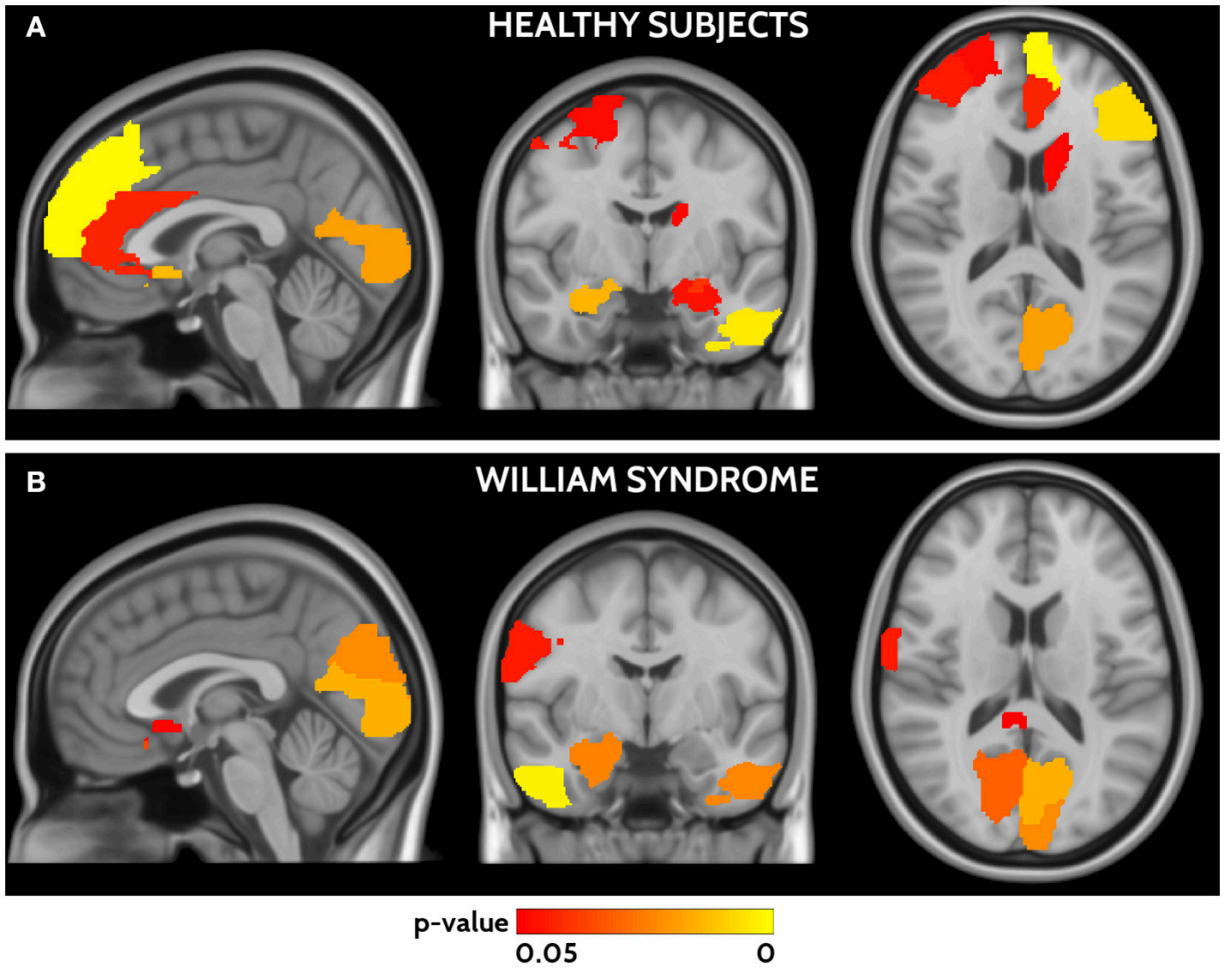
Areas of significant agreement between functional and structural connectivity for HCs **(A)** and WS **(B)** are shown in a red yellow scale according to the *p*-value. HCs show higher agreement in frontal, occipital, and temporal areas, whereas WS patients in parieto-occipital and temporal areas.

In HC frontal areas, hippocampi and amygdala, left temporal cortex, calcarine cortex, and caudate nucleus showed the highest agreement between functional and structural connectivity (Figure 4). In WS patients the highest agreement was found in temporal lobes, calcarine cortex, postcentral areas, cuneus, and small frontal areas (Figure 4). A detailed list of areas is reported in Supplementary Table 2. The direct comparison of WS patients and HC did not show any significant difference after correction for multiple-comparisons.

### Clinical data and correlation with connectivity measures

General cognitive competences were mildly impaired in the WS group (mean Full IQ 55.7), with non-verbal items being more significantly impaired than the verbal items (mean Performance and Verbal IQ were respectively 56.2 and 61.9; paired *t*-test: *p* = 0.02). Patients' general cognitive competences were quite homogeneously distributed (standard deviation: VIQ 7.7, PIQ 9.8, FIQ 8.3). The mean Full IQ for HC was 113.

To different extents, anxiety was a common problem among our WS participants. Only one male participant did not show anxiety behavior and 3 referred only a trend. All females had symptomatic anxiety problems as well as 3 males (out 7: 43%). We did not observe a significant relation between IQ and anxiety classes.

No significant correlation emerged between clinical measures (VIQ, PIQ, FIQ, psychopathological classes) and both structural or functional connectivity, except for a negative correlation (*r* = −0.97) between the presence of a generalized anxiety disorder in WS patients and the density of structural connections between the right superior occipital lobe and frontal supplementary motor area (Supplementary Figure 1).

## Discussion

Our group of late adolescents/young adults was homogeneous and displayed a typical WS profile, with overall mild cognitive impairment and anxious features in absence of other psychopathological symptoms. Their structural and functional brain features were compared with those of age—matched HCs (36) in order to identify pattern of altered connectivity among brain regions, and to test possible correlations with behavioral and cognitive measures (i.e., Anxiety, IQ).

DTI analysis revealed areas of lower FA both in deep (cerebellum, posterior limbs of internal capsules, in the splenium of corpus callosum) and subcortical regions (WM of the parieto-occipital region bilaterally) for WS patients compared to HC. Congruent with the peculiar cognitive profile, where the visual spatial impairment is prominent, we also found an asymmetrical involvement of parieto-occipital regions, with lower FA in the right hemisphere of the WS patient group. Previous literature has reported both higher and lower FA. Higher FA was observed in superior and inferior longitudinal fascicles, whereas lower FA values were reported in the posterior limbs of the internal capsules, middle and superior cerebellar peduncles, splenium of the corpus callosum, subcortical WM of the parieto-occipital regions bilaterally and in the uncinate fasciculus, which have been linked to the language, motor, visual spatial and face processing competences (37–39).

Altered fiber tract directionality, suggesting an increased ratio of longitudinally oriented fibers over transverse fibers, has been shown by Marenco et al. (20). Our analysis of structural connectivity, based on DTI data, expands this finding. The structural connectome revealed that the areas characterized by locally lower FA values showed abnormal connectivity in WS participants, mostly within the same hemisphere and to a lesser extent between hemispheres. The hyper-connectivity was observed to mainly affect antero-posterior pathways linking parieto-occipital with frontal regions, but was also detected, at a more local level, in temporal lobe and cerebellum.

The analysis of resting state fMRI showed an overall lower functional connectivity in our WS group compared to HCs in many significant brain networks. Among the few previous studies in literature, Vega et al. (21) described increased (compared to controls) between-network connectivity in the fronto-parietal DMN in their group of WS patients while Sampaio et al. (22) found a decreased FC in the posterior hub of the DMN. This modification has been putatively related with both immaturity of the brain and with the singularity of the WS behavioral phenotype. However, differences in FC in the DMN and/or fronto-parietal networks are not specific of WS and have been reported in other neurodevelopmental disorders [e.g., autism (40), ADHD (41), Down syndrome (42)] and may underlie an impaired ability to integrate information from distant brain regions into coherent distributed networks.

As to the correlation between areas of altered (functional or structural) connectivity and behavioral features (Anxiety and IQ), our findings show that the density of structural connections between supplementary motor and occipital areas in the right hemisphere was negatively correlated with the presence (clinical or sub-threshold) of anxiety disturbances; differently from Fan et al. (19), we did not find a relation between altered connectivity and overall cognitive competences. These may be well due to our low sample size, and this findings needs to be confirmed on larger samples.

Finally, when we compared the level of agreement between structural and functional connectivity according to the method proposed by Horn et al., we did not find significant differences between WS and HC. However, interesting trends emerged when considering the two groups separately. In HC, the highest agreement was observed in frontal and visual areas, which partially replicated the findings by Horn et al., Differently, in WS patients the highest level of functional and structural connectivity agreement was observed in posterior regions (parieto-occipital and temporal areas). Interestingly, these areas overlap with those found to be morphologically altered in the first study of WS brain architecture (18), and are behaviorally related to the main features of WS cognitive profile.

Though preliminary and limited by the low number of participants, our results from multimodal MR analysis highlight the presence of altered connectivity (structural and functional) in sensory perception and multisensory integration (mainly related with parieto-occipital networks), social-emotional processing (temporal tracts) and attention/control (frontal).

These findings might reflect a different, less efficient, “wiring” of the brain in WS. Hoeft et al. (37) observed less distinct or segregated functional connectivity in WS compared to HC, and concluded that “more is not always better.” Similarly, here we found that areas with increased structural connections were also characterized by impaired functional connectivity. In typical development, modularity decreases and global efficiency increases along with age; structural networks become more globally and less locally efficient with age, but the opposite is true for functional networks.

The alteration of the structural and functional connectivity in occipito-parietal, frontal and temporal lobes could be linked to the impairment in dorsal stream functions that may occur in WS as well as in many other clinical conditions (43–45). As suggested by Shore et al. (46), low-level perceptual processes can have a cascading effect on social cognition. In a developmental perspective the connections in the occipito-parietal network take place earlier than in other brain regions. Therefore, an impaired (or modified) perceptual integration network can potentially affect and limit other connected networks such as frontal and temporal ones whose organization continues up to young adultness. This could help to explain the peculiarity of the WS behavioral profile, where the visual spatial impairment together with multiple difficulties in other domains (such as memory and executive functions) is crucial, but where anxiety and social behavior play a relevant role.

Some limitations of this study must be acknowledged. The main limitation of our work is the small sample size that prevents the generalization of our results. The young adults with WS who freely adhered to the research proposal were highly motivated and very collaborative, given the necessity to cope with the MRI exam and noise for a relatively long time. However, the sample-size was relatively small due to the exclusion of low-functioning/less cooperative subjects and this prevented us from extending conclusions to more impaired patients. This bias in the recruitment is shared—though seldom explicitly recognized—with most other studies. Another limitation regarding our cohort is the relatively wide age-range of patients, from late adolescents to young adults. The effects of late development of frontal regions through early adulthood have not been taken into account and should be matter of future investigations. Also an analysis of inter-network connectivity could improve our knowledge about the reorganization of brain circuits in WS.

Among the number of studies about the role of the genes of the WSCR in the clinical and behavioral WS phenotype, many focused on the characteristics of the visual spatial impairment, anxiety and social behavior in atypical deletions [see for instance (47, 48)]. Our cohort of WS patients carried the typical deletion. For this reason, we cannot shed new light on the role of specific genes, but we can confirm how the typical deletion of the WSCR is characterized by a behavioral phenotype linked to a peculiar brain organization.

More studies and larger samples are needed to confirm and better detail our findings; however, we demonstrated that simultaneous assessment of functional and structural connectivity in WS is feasible and that these techniques seems to be capable of providing further insights in brain organization, and therefore to improve our therapeutic and rehabilitation approaches.

## Data availability statements

Preprocessed MRI data will be made available to researchers upon requests directed to Dr. Filippo Arrigoni (filippo.arrigoni@bp.lnf.it).

## Author contributions

CG, FA, and RB conceptualized and designed the study, drafted the initial manuscript, and approved the final manuscript as submitted. AN and DP carried out the fMRI analyses, and approved the final manuscript as submitted. ADL and AL carried out dMRI analyses, reviewed and revised the manuscript, and approved the final manuscript as submitted. AD critically reviewed the manuscript, and approved the final manuscript as submitted.

### Conflict of interest statement

The authors declare that the research was conducted in the absence of any commercial or financial relationships that could be construed as a potential conflict of interest.

## Abbreviations

dMRI: Diffusion MRI
DMN: Default Mode Network
DTI: Diffusion Tensor Imaging
FA: Fractional anisotropy
FC: Functional connectivity
fMRI: Functional MRI
GM: Gray matter
HC: Healthy controls
ICA: Independent component analysis
WM: White matter
WS: Williams syndrome
WSCR: WS critical region.
