# Supplementary material for: A Different Brain: Anomalies of Functional and Structural Connections in Williams Syndrome
Source: Front Neurol. 2018 Sep 13;9:721. doi: 10.3389/fneur.2018.00721 (PMC6146099; doi:10.3389/fneur.2018.00721)

**SUPPLEMENTARY MATERIAL**

Title: A different brain: Anomalies of functional and structural connections in Williams syndrome

Authors: Chiara Gagliardi, Filippo Arrigoni, Andrea Nordio, Alberto De Luca, Denis Peruzzo, Alice Decio, Alexander Leemans, Renato Borgatti

Journal: Frontiers in Neurology – Applied Neuroimaging

Corresponding Author: Filippo Arrigoni, MD. Neuroimaging Lab, Scientific Institute IRCCS Eugenio Medea

Email address: filippo.arrigoni@lanostrafamiglia.it

**Supplementary Table 1:** Structural connections showing significant differences between WS and HCs

|  |  |  |
| --- | --- | --- |
| **Node A** | **Node B** | **T-value** |
| Calcarine Right | Para Hippocampus Right | 6,09 |
| Cerebellum 7b Right | Cerebellum Crus1 Right | 3,65 |
| Cingulum Mid Right | Frontal Inferiorerior Orbit Right | 4,03 |
| Cuneus Left | Precentral Right | 2,85 |
| Cuneus Left | Frontal Superior Left | 4,44 |
| Cuneus Left | Superior Motor Area Left | 3,22 |
| Cuneus Left | Superior Motor Area Right | 4,76 |
| Cuneus Left | Frontal Superior Medial Left | 4,65 |
| Cuneus Left | Cingulum Anterior Left | 4,01 |
| Cuneus Left | Cingulum Anterior Right | 3,55 |
| Cuneus Right | Precentral Left | 3,11 |
| Cuneus Right | Frontal Superior Right | 3,31 |
| Cuneus Right | Frontal Mid Right | 2,72 |
| Cuneus Right | Frontal Inferior Tri Right | 2,68 |
| Cuneus Right | Superior Motor Area Left | 4,44 |
| Cuneus Right | Superior Motor Area Right | 3,80 |
| Cuneus Right | Frontal Superior Medial Right | 4,75 |
| Cuneus Right | Cingulum Anterior Right | 4,30 |
| Cuneus Right | Cingulum Mid Right | 4,43 |
| Cuneus Right | Hippocampus Right | 2,56 |
| Cuneus Right | Paracentral Leftobule Left | 4,04 |
| Occipital Superior Left | Precentral Right | 2,14 |
| Occipital Superior Left | Frontal Superior Left | 3,21 |
| Occipital Superior Left | Frontal Mid Left | 4,08 |
| Occipital Superior Left | Frontal Inferior Oper Left | 3,85 |
| Occipital Superior Left | Superior Motor Area Left | 2,87 |
| Occipital Superior Left | Superior Motor Area Right | 3,13 |
| Occipital Superior Left | Frontal Superior Medial Left | 3,85 |
| Occipital Superior Right | Precentral Right | 3,35 |
| Occipital Superior Right | Frontal Superior Right | 2,50 |
| Occipital Superior Right | Frontal Mid Right | 3,37 |
| Occipital Superior Right | Frontal Inferior Tri Right | 3,85 |
| Occipital Superior Right | Frontal Inferior Orbit Right | 2,66 |
| Occipital Superior Right | Superior Motor Area Right | 2,52 |
| Occipital Superior Right | Cingulum Mid Right | 3,26 |
| Occipital Superior Right | Heschl Right | 4,96 |
| Occipital Superior Right | Temporal Superior Right | 4,37 |
| Olfactory Left | Frontal Superior Orb Left | 5,39 |
| Paracentral Leftobule Right | Cingulum Anterior Left | 3,20 |
| Parietal Superior Left | Frontal Superior Left | 3,34 |
| Parietal Superior Left | Frontal Mid Left | 2,47 |
| Parietal Superior Left | Superior Motor Area Left | 3,43 |
| Parietal Superior Right | Frontal Superior Left | 3,59 |
| Postcentral Left | Frontal Mid Left | 5,32 |
| PreCuneus Left | Frontal Superior Left | 3,43 |
| PreCuneus Left | Superior Motor Area Left | 2,76 |
| PreCuneus Right | Superior Motor Area Right | 4,09 |
| PreCuneus Right | Frontal Superior Medial Right | 2,81 |
| PreCuneus Right | Cuneus Right | 5,31 |
| Vermis 8 | Cerebellum 4 5 Right | 6,30 |

**Supplementary Table 2:** Mean agreements coefficients for structural and functional connectivity in HC and WS. AAL ROIs where evaluated, excluding cerebellum regions. In bold, ROIs where the difference between HC and WS was significant (p < 0.05).

| **ROI** | **Agreement HC**  **Mean (sd)** | **Agreement WS**  **Mean (sd)** |
| --- | --- | --- |
| Precentral_L | 0,46 (0,13) | 0,52 (0,06) |
| **Precentral_R** | **0,43 (0,12)** | **0,56 (0,08)** |
| Frontal_Sup_L | 0,55 (0,08) | 0,56 (0,07) |
| Frontal_Sup_R | 0,57 (0,09) | 0,58 (0,06) |
| Frontal_Sup_Orb_L | 0,58 (0,09) | 0,61 (0,08) |
| Frontal_Sup_Orb_R | 0,55 (0,09) | 0,61 (0,09) |
| Frontal_Mid_L | 0,54 (0,10) | 0,56 (0,08) |
| Frontal_Mid_R | 0,55 (0,05) | 0,54 (0,10) |
| Frontal_Mid_Orb_L | 0,54 (0,07) | 0,56 (0,05) |
| Frontal_Mid_Orb_R | 0,51 (0,05) | 0,55 (0,08) |
| Frontal_Inf_Oper_L | 0,53 (0,07) | 0,55 (0,05) |
| Frontal_Inf_Oper_R | 0,51 (0,05) | 0,55 (0,08) |
| Frontal_Inf_Tri_L | 0,57 (0,05) | 0,56 (0,04) |
| Frontal_Inf_Tri_R | 0,54 (0,06) | 0,58 (0,09) |
| Frontal_Inf_Orb_L | 0,52 (0,06) | 0,56 (0,08) |
| Frontal_Inf_Orb_R | 0,49 (0,09) | 0,52 (0,09) |
| Rolandic_Oper_L | 0,52 (0,10) | 0,51 (0,04) |
| Rolandic_Oper_R | 0,47 (0,09) | 0,55 (0,05) |
| Supp_Motor_Area_L | 0,52 (0,09) | 0,51 (0,10) |
| **Supp_Motor_Area_R** | **0,48 (0,07)** | **0,56 (0,04)** |
| Olfactory_L | 0,58 (0,07) | 0,61 (0,10) |
| Olfactory_R | 0,63 (0,09) | 0,57 (0,11) |
| Frontal_Sup_Medial_L | 0,57 (0,03) | 0,55 (0,07) |
| Frontal_Sup_Medial_R | 0,55 (0,06) | 0,55 (0,06) |
| Frontal_Med_Orb_L | 0,51 (0,07) | 0,55 (0,04) |
| Frontal_Med_Orb_R | 0,50 (0,07) | 0,56 (0,05) |
| Rectus_L | 0,52 (0,08) | 0,58 (0,07) |
| Rectus_R | 0,53 (0,07) | 0,61 (0,08) |
| Insula_L | 0,47 (0,07) | 0,50 (0,07) |
| Insula_R | 0,49 (0,08) | 0,52 (0,10) |
| Cingulum_Ant_L | 0,56 (0,07) | 0,55 (0,09) |
| Cingulum_Ant_R | 0,52 (0,09) | 0,56 (0,08) |
| Cingulum_Mid_L | 0,44 (0,07) | 0,48 (0,09) |
| Cingulum_Mid_R | 0,42 (0,06) | 0,48 (0,11) |
| Cingulum_Post_L | 0,48 (0,02) | 0,50 (0,06) |
| **Cingulum_Post_R** | **0,53 (0,03)** | **0,61 (0,10)** |
| Hippocampus_L | 0,55 (0,06) | 0,55 (0,07) |
| Hippocampus_R | 0,60 (0,08) | 0,59 (0,05) |
| ParaHippocampal_L | 0,55 (0,10) | 0,57 (0,06) |
| ParaHippocampal_R | 0,53 (0,09) | 0,61 (0,07) |
| Amygdala_L | 0,58 (0,09) | 0,57 (0,08) |
| Amygdala_R | 0,52 (0,10) | 0,59 (0,10) |
| Calcarine_L | 0,59 (0,08) | 0,63 (0,08) |
| Calcarine_R | 0,54 (0,06) | 0,59 (0,06) |
| **Cuneus_L** | **0,51 (0,09)** | **0,60 (0,06)** |
| Cuneus_R | 0,48 (0,07) | 0,55 (0,05) |
| **Lingual_L** | **0,42 (0,04)** | **0,56 (0,04)** |
| **Lingual_R** | **0,44 (0,08)** | **0,56 (0,05)** |
| **Occipital_Sup_L** | **0,47 (0,07)** | **0,58 (0,05)** |
| Occipital_Sup_R | 0,49 (0,08) | 0,56 (0,07) |
| **Occipital_Mid_L** | **0,50 (0,04)** | **0,56 (0,06)** |
| Occipital_Mid_R | 0,46 (0,07) | 0,52 (0,06) |
| Occipital_Inf_L | 0,49 (0,08) | 0,54 (0,09) |
| Occipital_Inf_R | 0,49 (0,10) | 0,54 (0,11) |
| Fusiform_L | 0,47 (0,09) | 0,56 (0,06) |
| **Fusiform_R** | **0,47 (0,10)** | **0,57 (0,05)** |
| Postcentral_L | 0,47 (0,14) | 0,53 (0,08) |
| **Postcentral_R** | **0,46 (0,13)** | **0,59 (0,06)** |
| **Parietal_Sup_L** | **0,44 (0,08)** | **0,55 (0,08)** |
| Parietal_Sup_R | 0,43 (0,08) | 0,49 (0,04) |
| Parietal_Inf_L | 0,47 (0,08) | 0,50 (0,11) |
| Parietal_Inf_R | 0,52 (0,03) | 0,48 (0,05) |
| **SupraMarginal_L** | **0,43 (0,04)** | **0,53 (0,08)** |
| **SupraMarginal_R** | **0,45 (0,05)** | **0,51 (0,05)** |
| Angular_L | 0,45 (0,08) | 0,50 (0,09) |
| Angular_R | 0,48 (0,07) | 0,47 (0,04) |
| **Precuneus_L** | **0,48 (0,06)** | **0,54 (0,04)** |
| Precuneus_R | 0,50 (0,08) | 0,55 (0,06) |
| Paracentral_Lobule_L | 0,46 (0,10) | 0,53 (0,07) |
| Paracentral_Lobule_R | 0,52 (0,10) | 0,54 (0,10) |
| Caudate_L | 0,56 (0,07) | 0,54 (0,04) |
| Caudate_R | 0,51 (0,10) | 0,51 (0,08) |
| Putamen_L | 0,54 (0,07) | 0,53 (0,10) |
| Putamen_R | 0,55 (0,09) | 0,51 (0,11) |
| Pallidum_L | 0,58 (0,14) | 0,59 (0,10) |
| Pallidum_R | 0,58 (0,12) | 0,59 (0,07) |
| Thalamus_L | 0,45 (0,07) | 0,46 (0,10) |
| Thalamus_R | 0,45 (0,08) | 0,43 (0,11) |
| Heschl_L | 0,48 (0,14) | 0,54 (0,08) |
| Heschl_R | 0,49 (0,18) | 0,55 (0,07) |
| Temporal_Sup_L | 0,40 (0,07) | 0,46 (0,08) |
| **Temporal_Sup_R** | **0,44 (0,06)** | **0,57 (0,11)** |
| Temporal_Pole_Sup_L | 0,51 (0,09) | 0,57 (0,08) |
| Temporal_Pole_Sup_R | 0,50 (0,07) | 0,57 (0,08) |
| Temporal_Mid_L | 0,44 (0,07) | 0,48 (0,06) |
| Temporal_Mid_R | 0,46 (0,07) | 0,54 (0,07) |
| Temporal_Pole_Mid_L | 0,53 (0,11) | 0,62 (0,07) |
| Temporal_Pole_Mid_R | 0,53 (0,10) | 0,60 (0,07) |
| Temporal_Inf_L | 0,58 (0,05) | 0,64 (0,10) |
| **Temporal_Inf_R** | **0,53 (0,07)** | **0,62 (0,05)** |

Supplementary Figure 1. Scatterplot of the significant correlation between anxiety and structural connections between occipital and frontal areas.


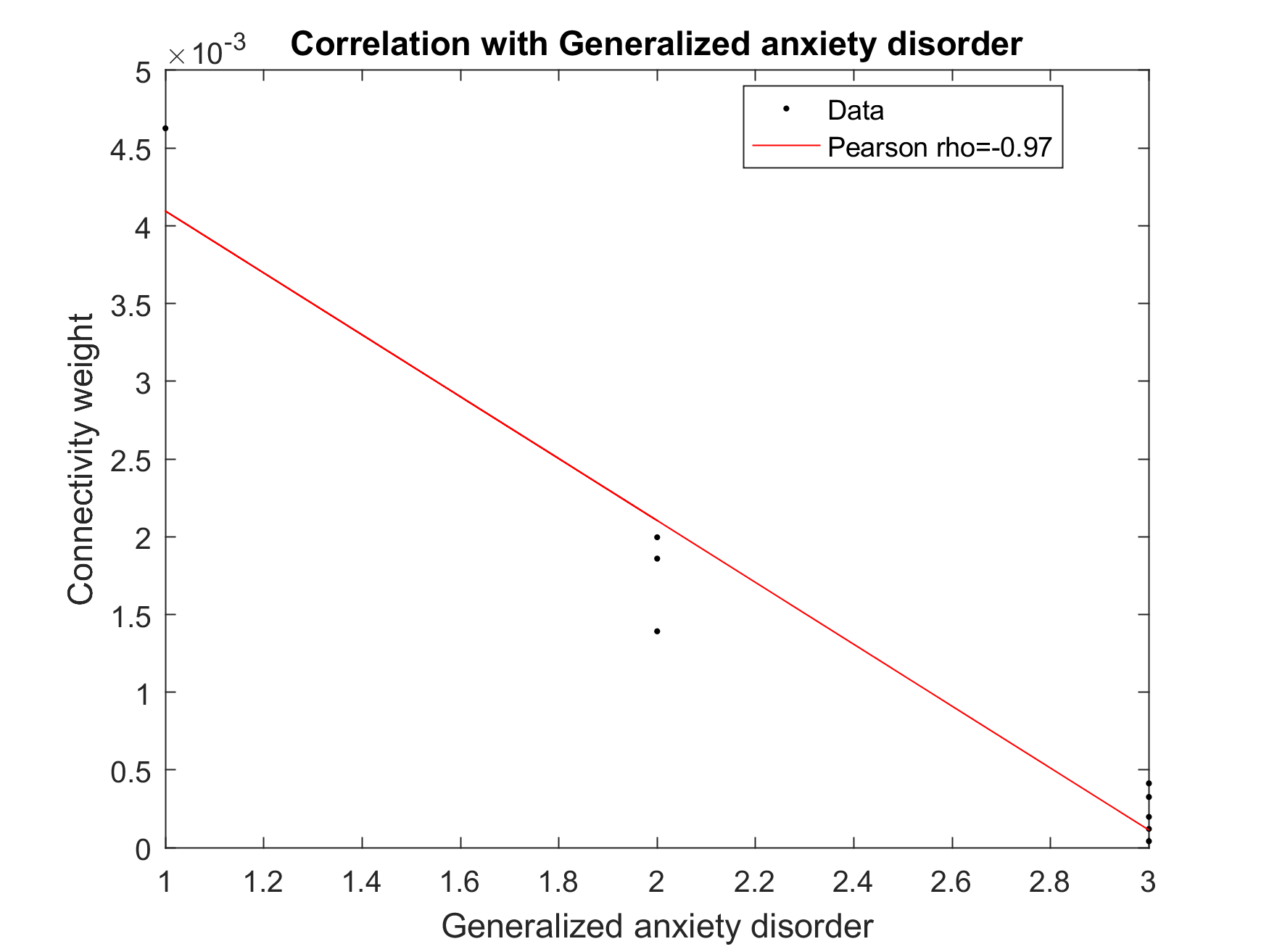

Supplement: Supplementary file 1 [file Data_Sheet_1.docx]
